# Negative Regulation of Interferon-β Gene Expression during Acute and Persistent Virus Infections

**DOI:** 10.1371/journal.pone.0020681

**Published:** 2011-06-03

**Authors:** Junqiang Ye, Tom Maniatis

**Affiliations:** Department of Molecular and Cellular Biology, Harvard University, Cambridge, Massachusetts, United States of America; University of California, San Francisco, United States of America

## Abstract

The production of type I interferons (IFNs) in response to viral infections is critical for antiviral immunity. However, IFN production is transient, and continued expression can lead to inflammatory or autoimmune diseases. Thus, understanding the mechanisms underlying the negative regulation of IFN expression could lead to the development of novel therapeutic approaches to the treatment of these diseases. We report that the transcription factor IRF3 plays a central role in the negative regulation of interferon-β (IFNβ) expression during both acute and persistent (chronic) virus infections. We show that the degradation of IRF3 during acute infections, rather than the activation of transcriptional repressors, leads to the down regulation of IFNβ expression. We also show that the block to IFNβ expression in mouse embryonic fibroblasts that are persistently infected with Sendai virus (SeV) correlates with the absence of transcriptionally active IRF3. Remarkably, ongoing protein synthesis and viral replication are required to maintain repression of the IFNβ gene in persistently infected cells, as the gene can be activated by the protein synthesis inhibitor cycloheximide, or by the antiviral drug ribavirin. Finally, we show that the SeV V protein inhibits IRF3 activity in persistently infected cells. Thus, in conjunction with the known interference with STAT1 by the SeV C protein, both IFN activation and its signaling pathways are blocked in persistently infected cells. We conclude that the transcription factor IRF3 is targeted for turnover and inactivation through distinct mechanisms from both the host cells and virus, leading to the inhibition of IFNβ gene expression during acute and persistent viral infections. These observations show that IRF3 plays a critical role, not only in the activation of the IFNβ gene, but also in the controlling the duration of its expression. (284 words)

## Introduction

Virus infection induces the transient expression of type I interferons (IFNs) in virtually every cell type [1]. Secreted IFNs bind to cell surface receptors in both the infected and surrounding cells to induce antiviral activities encoded by interferon stimulated genes (ISGs). IFNs also coordinate the activation of the adaptive immune system, which is necessary to control the spread of infection [1], [2], [3].

Regulation of interferon-β (IFNβ) gene expression has been extensively studied [4], [5], and the regulatory sequences, critical transcription factors and components of the virus-induced signaling pathway identified [6]. Viral RNA is detected by RIG-I and MDA5 in most cells [7]. Both proteins undergo a conformational change upon binding to a 5′-triphosphate panhandle RNA or long double stranded RNAs (dsRNAs) associated with virus infection and replication [8], [9]. This conformational change leads to homodimerization of the RNA sensors, and signal transmission through a critical adaptor protein MAVS located on the mitochondrial membrane [10], [11]. This interaction requires caspase-recruiting domains (CARD) on both the RNA sensors and MAVS [11], [12], [13], [14]. Once engaged with RIG-I or MDA5, the MAVS complex recruits the downstream adaptor proteins TRAF3 and TRAF6, and the kinases TBK1 [12], [15] and IKKα/β [11], [13], leading to the activation of the critical transcription factors IRF3/7 and NFκB. Phosphorylated IRF3/7 and NFκB translocate into the nucleus, and together with cJUN/ATF2 and the transcriptional coactivators CBP/p300 form an enhanceosome complex upstream of the IFNβ gene promoter [4]. Chromatin remodeling factors and the basic transcription machinery are then recruited to drive the expression of the gene [16].

The production of IFN is essential for countering virus infections, but IFN gene expression must be tightly regulated. The continued expression of IFN is toxic, and over-expression can contribute to inflammatory and autoimmune diseases [17], [18], [19]. Thus, tight regulation of the level and duration of IFN expression is necessary to mount a strong antiviral response on the one hand, while preventing the negative effects of IFN overproduction on the other.

A number of proteins that negatively regulate IFN expression have been identified, and virtually every component in the virus induction signaling pathway is controlled by either host or viral proteins. For example, the RIG-I protein is down regulated by the host protein RFN125, CYLD, NLRC5, Casein kinase II and other kinases [20], [21], [22], [23], [24], [25]; the MAVS protein is also negatively regulated by the host proteins NLRX1 and PCBP2 [26], [27], and is cleaved from the mitochondria surface by the NS3/4 protease of the hepatitis C virus (HCV) [28]. Moreover, the adaptor proteins TRAF3 and TRAF6 are targeted by the cellular proteins DUBA and A20 [29], [30], and TBK1 is sequestered by SIKE [31]. The transcription activator IRF3 is under negative regulation by host protein Pin1 and MafB, and HIV accessory proteins VPR and Vif [32], [33], [34], and the p65 subunit of NFκB is targeted for degradation by PDLIM2 [35]. All of these proteins suppress IFNβ gene expression.

A common feature of these negative regulators of the virus infection signaling pathway is that their ability to inhibit or enhance expression of IFNβ correlates with their increased or reduced expression, respectively. However, it is important to note that none of these factors are required to turn off IFNβ expression following virus infection. Thus, the mechanisms of post-induction termination of IFNβ expression are largely unknown. The IFNβ gene is transiently expressed in response to viral infection. In most cultured cells, IFNβ gene transcripts are typically detected within 3–6 hrs after infection, peak at 9–12 hrs and return to base line by 24 hrs [36], [37]. In animals, expression of the IFNβ gene is also turned-off a few days after virus infection [38], [39]. Thus, it appears that switching off IFNβ gene expression during the time course of virus infection is an integral part of the innate immunity regulatory mechanism.

Early studies established that the post-induction turn-off of IFNβ gene expression is primarily at the level of transcription and not mRNA turnover [37]. However, the question of whether the termination of IFNβ transcription is due to the inactivation of transcriptional activators or the induction of repressors or the combination of both has not been answered. We have therefore investigated the mechanisms of post-induction turn-off of IFNβ gene expression during acute virus infection.

Viruses have evolved the ability to suppress IFN gene expression to avoid the antiviral response and either continue lytic growth or establish a persistent (chronic) infection. For example, both the hepatitis C virus (HCV) [40], [41] and the human immunodeficiency virus (HIV) [42], [43] establish persistent infections, and therefore pose major challenges to human health. Similarly, lymphocytic choriomeningitis virus (LCMV) can establish persistent infection in the mouse central nervous and immune systems [44], [45], and Sendai virus is also able to establish persistent infections in mouse respiratory tissues [46]. The infecting virus enters the persistent state by either high replication or latency. The former is associated with active viral replication in infected hosts, and virus products inhibit the host immune responses [47]. In the case of the latent response the virus persists in a quiescent state, but can reactivate replication under appropriate circumstances [47]. Persistent viral infection poses a great threat to human health, as uncontrolled viral replication will exhaust host resources and lead to cell death; the impaired host immune response also makes infected individuals highly vulnerable to opportunistic infection [47].

The control of virus replication in persistently infected individuals has been the focus of many studies, but the role of IFN in persistent infection has not been determined. In principle, the antiviral effects of IFN should suppress persistent infections. In fact, persistently infected HCV patients have been shown to benefit from IFN treatment [48]. However, when HIV infected patients were treated with IFN no effect on virus replication was observed, and IFN was detected in the serum of HIV patients not treated with IFN [49], [50]. In addition, prolonged IFN production in AIDS patients appears to contribute to the transition from persistent to pathogenic HIV infection [50], [51]. Consistent with this possibility, recent studies showed that a primary difference between non-pathogenic and pathogenic AIDS virus infection is the duration of the expression of IFN and the induced ISGs: a sustained expression of these genes was observed in pathogenic infections, in contrast to a transient expression in non-pathogenic infections [38], [39].

Here we present the results of a study of the negative regulation of IFN expression during acute SeV infection and in a cellular model of SeV persistent infection. In both cases we show that the transcription factor IRF3 is a key protein targeted for negative regulation of IFNβ expression. Our studies point to the regulation of IRF3 as a critical factor in the prevention of virus-induced diseases.

## Results

### The role of IRF3 degradation in the post-induction turn-off of IFNβ expression

While the activation of IFNβ gene expression by virus infection is well understood, its turn-off is not. Previous studies have shown that the post-induction decrease in IFNβ gene expression occurs at the level of transcription [37]: nuclear run-on assays measuring the transcription rate revealed that the decrease of IFNβ mRNA coincides with the termination of transcription. Treatment of virus-infected cells with cycloheximide (CHX) (an inhibitor of protein synthesis) prevents the turn-off of IFNβ transcription, and also stabilizes the steady state level of the IFNβ mRNA. Two models have been proposed to explain this data: In the repressor model, newly synthesized repressor(s) (whose synthesis is blocked by CHX treatment) competes with transcriptional activators for binding to the IFNβ promoter, and when bound maintains the promoter in an “off” state. In the second model the transcriptional activators required for IFNβ gene expression are down regulated, and the required regulatory proteins cannot be synthesized in the presence of CHX. Which of these models is correct is not known.

We first studied whether the inactivation of transcription factors is the primary mechanism for IFNβ turn-off. We monitored the expression of key transcription factors during the normal time course of virus infection, as well as infection in the presence of CHX, which prevents the post-induction turn-off of IFNβ gene expression.

**Figure 1:**
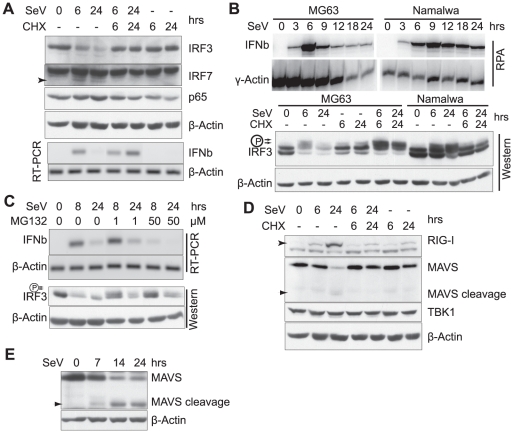
The degradation of IRF3 correlates with the turn-off of IFNβ expression. **A**. Virus-induced IRF3 degradation is blocked by CHX treatment. MEFs were treated with SeV and/or CHX for the indicated times. Half of the samples were lysed for total protein extracts and subjected to western blot analysis, probing for IRF3, IRF7, p65 and β-Actin. RNA was extracted from the other half of the sample. cDNAs were prepared and the expression of IFNβ and β-Actin monitored by RT-PCR. **B**. CHX blocks virus-induced degradation of IRF3 in human cells. Human MG63 or Namalwa cells were treated as in A, total protein was prepared and analyzed for the expression of IRF3 and β-Actin. RNase protection assays (RPA) were conducted to monitor the expression of IFNβ and γ-Actin mRNA (top panel). **C**. The proteosome inhibitor MG132 only partially inhibits the virus-induced degradation of IRF3. MEFs were infected with SeV in the presence or absence of 1 µM or 50 µM of MG132 for the indicated times, cells were harvested for analysis of IRF3 and β-Actin protein expression or IFNβ and β-Actin mRNA levels. **D**. The MAVS protein is cleaved and degraded in SeV infected MEFs. MEFs were treated the same as in A, and the level of RIG-I, MAVS, and TBK1 proteins monitored using the appropriate antibodies. **E**. Cleavage and degradation of MAVS revealed by another antibody. MEFs infected by SeV were lysed at different time points, and subjected to western blot analysis with another anti-mouse MAVS antibody.

IRF3, which is an essential transcriptional activator of the IFNβ gene, has been shown to undergo virus-induced phosphorylation and subsequent degradation [52], [53]. However, the nature of the degradation has not been fully defined, and its role in IFNβ turn-off has not been established. We first monitored the IRF3 protein levels in mouse embryonic fibroblasts (MEF) infected with Sendai virus (SeV) in the presence or absence of CHX. As previously shown [52], [53], IRF3 undergoes virus-induced degradation, and after 24 hrs infection, relatively little IRF3 protein could be detected (Fig. 1A). By contrast, in the presence of CHX the level of IRF3 did not change during the time course of infection (Fig. 1A). The levels of IRF3 in the absence or presence of CHX correlate well with the turn-off or continued expression of the IFNβ gene (Fig. 1A, bottom panel). Thus, the degradation of IRF3 during virus infection is likely to play a key role in the turn-off of IFNβ expression. Additional experiments showed that the level of the NFκB p65 subunit was unaffected during virus infection, and the level of IRF7 was induced 6 hrs after virus infection (the IRF7 antibody cross-reacts with another protein, IRF7 is seen as a more rapidly migrating faint band). Only a slight decrease in IRF7 protein was observed 24 hr post-infection, in contrast to the major degradation of IRF3 (Fig. 1A). Thus, IRF3 appears to be unique among the transcription factors required for IFN expression with respect to post-induction turnover.

To exclude the possibility that the post-induction degradation of IRF3 occurs only in murine cells, we also monitored the levels of IRF3 in human cells after SeV infection. As shown in Figure 1B, CHX abolished the virus-induced degradation of IRF3 in human MG63 cells, and as a result, sustained expression of IFNβ was observed [37]. Interestingly, treatment of SeV infected cells with CHX induced much higher levels of phosphorylated IRF3 compared to that observed with virus or CHX alone (Fig. 1B). This increase could contribute to the “super-induction” of IFN reported in earlier studies [37]. Importantly, IRF3 turn-over was not observed after 24 hrs of infection in the presence of CHX, in direct contrast to the degradation of IRF3 observed in cells infected by SeV in the absence of CHX (Fig. 1B). Thus, CHX inhibits the virus-induced degradation of IRF3 protein in both MEFs and MG63 cells, and as a result, IFNβ expression continues. By contrast, IFNβ turn-off is slower in human Namalwa cells (Fig. 1B top panel). We note that virus infection also induced some degradation of IRF3 in Namalwa cells, but, the level of the phosphorylated IRF3 remained high as late as 24 hrs post-infection (Fig. 1B). As with the other cell lines, CHX abolished IRF3 turnover. Taken together with previous observations [37], these data strongly suggest that the degradation of phosphorylated (activated) IRF3 protein is a primary mechanism for the transcriptional turn-off of the IFNβ gene during acute virus infections.

In previous studies the virus-induced degradation of IRF3 was shown to depend on the ubiquitin-proteosome system [52], [53]. We therefore tested the effects of the proteosome inhibitor MG132 on the degradation of IRF3 and subsequent IFNβ turn-off. Consistent with previous reports, MG132 treatment did lead to accumulation of phosphorylated IRF3 in MEFs (Fig. 1C). Surprisingly, however, the degradation of IRF3 was only partially inhibited by MG132 treatment. The effects were stronger with shorter SeV infection (8 hrs post infection, Fig. 1C). However, significant degradation of IRF3 was also observed at 24 hours after infection (Fig. 1C), and as a result, the expression of the IFNβ gene was still turned-off (Fig. 1C top panel). Higher concentrations of MG132 (50 µM) inhibited the activation of the IFNβ gene (Fig. 1C), most likely due to blocking the degradation of IKBα, the inhibitor of NFκB [54], as demonstrated by its inhibition of TNF induced IKBα degradation (Fig. S1A). Significant degradation of IRF3 was similarly observed 24 hours after SeV infection in the presence of lactacystin, another specific proteosome inhibitor (Fig. S1B). Thus, our data clearly show that the ubiquitin-proteosome system is not sufficient to completely inactivate IRF3. Other inducible proteases (blocked by CHX treatment) may also be required for the complete degradation of IRF3.

### The PRDI-BF1 and PRDII-BF1 repressors are not required for post-induction IFNβ turn-off

Previous studies implicated two proteins, PRDI-BF1 and PRDII-BF1 in the post-induction repression of IFNβ expression [36], [55]. The expression of both proteins is induced by virus infection, and the kinetics of their induction is delayed compared to that of the IFNβ gene [36], [55]. They both bind specifically to the IFNβ promoter, and both can function as repressors *in vivo* [56], [57], [58]. Transient over-expression of either repressor in cultured cells potently inhibited IFNβ reporter expression, strongly suggesting a role in post-induction repression of IFN expression [36], [55], [59]. However, contrary to expectation, we found that reducing or eliminating the expression of either PRDI-BFI or PRDII BF-1 had little if any effect on the induction kinetics of the IFNβ gene. Specifically, knocking-down the expression of PRDI-BF1 in human MG63 cells by siRNA (Fig. S2), or completely knocking-out either PRDI-BF1 or PRDII-BF1 expression in MEFs did not alter the kinetics of IFNβ expression in response to virus infection (Fig. S3). In addition, Knocking-down the expression of PRDII-BF1 in PRDI-BF1 knockout MEFs did not affect the kinetics of IFNβ turn-off (Fig. S4A–S4C). Thus, we conclude that neither PRDI-BF1 nor PRDII-BF1 is required for the post-induction repression of IFNβ expression. Although we cannot exclude the possibility that yet to be identified repressors play a role in IFNβ turn-off, or that these repressors function in other cell types, it appears that the inactivation of the IRF3 protein alone is the mechanism for shutting off IFNβ gene expression during virus infection.

### SeV-induced cleavage and degradation of MAVS in MEFs

We also explored the possibility that signaling components in the IFNβ induction pathway are degraded, preventing the continuous activation of critical transcription factors, including IRF3. Monitoring the expression of upstream signaling components in MEFs during SeV infection revealed distinct expression patterns for RIG-I, MAVS and TBK1 proteins (Fig. 1D). There was little change in the level of TBK1 protein during infection. In contrast, the RIG-I protein was strongly induced during the time course of infection, as expected for an interferon inducible gene. Strikingly, the level of the MAVS protein decreased over time, and a 55KD band, likely a cleavage product of MAVS, appeared at a later time point after viral infection (Fig. 1D). This was better shown using a more sensitive MAVS antibody [60]: the level of full length MAVS protein decreased between 7 and 14 hrs, and then remained constant. Coincidentally, an induced 55KD band appeared around 7 hrs, increased in intensity 14 hrs after infection and then remained constant (Fig. 1E). Since the decreased level of full length MAVS protein correlates with the appearance of the new band, it is likely that this band is a cleavage product of MAVS. Virus-induced degradation and cleavage of MAVS protein was also observed in the murine Raw264.7 cell line (Fig. S5A). Interestingly, CHX blocked SeV-induced MAVS cleavage (Fig. 1D). Considering that IFN continues to be produced in the presence of CHX, this observation shows that MAVS cleavage is likely to be a post-induction event not required for the activation of the IFNβ gene. However, we investigated the function of virus-induced MAVS cleavage. Experiments with MAVS deficient MEFs reconstituted with a construct encoding mutant MAVS protein that is not cleavable shows that the cleavage of MAVS is not required for IFNβ turn-off (Fig. S6). The same experiments also showed that the cleavage of MAVS is not required for its degradation (Fig. S6). Since CHX abolished post-induction IFNβ turn-off without elimination of MAVS degradation (Fig. 1A, 1D), it is likely that the degradation of MAVS is dispensable for IFNβ turn-off. Nevertheless, the cleavage and degradation of MAVS appears to provide another mechanism to prevent the continuous activation of downstream factors.

We also note that the E3 ligase Itch, which has been reported to be responsible for the SeV-induced MAVS degradation [27], does not appear to be involved in the regulation of either the cleavage or the degradation of MAVS in our hands (Fig. S5B).

### Establishment of Sendai virus persistent infection in cultured cells

In the course of studying SeV-induced IFNβ expression kinetics, we observed dramatically different fates of infected cells. Viral infection of most cells leads to rapid cell death: almost 100% of the cells die after 24 hrs infection with L929 and Raw264.7 cells; while MG63 and Hela cells survive slightly longer but eventually die. Interestingly, the growth of Namalwa cells and MEFs does not appear to be affected by virus infection. To investigate this phenomenon we attempted to maintain infected cells in culture, and monitored the virus production and IFNβ expression. Remarkably, the virus load in Namalwa cells gradually decreased with time, and IFNβ gene expression also decreased (as mentioned above, Namalwa cells have a slow IFNβ turn-off rate). By day 19 post-infection, viral particles were difficult to detect. These observations suggest that cultured Namalwa cells can eventually clear the virus.

**Figure 2:**
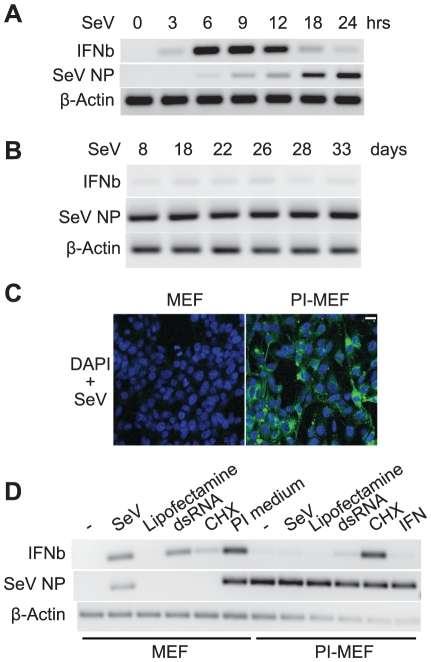
Establishment of persistent SeV infection in MEFs. **A**. The kinetics of IFNβ expression and virus replication during acute SeV infection. MEFs were infected with SeV and incubated for the indicated times, total RNA was prepared, and the expression of IFNβ, SeV NP and β-Actin monitored by RT-PCR. **B**. SeV establishes a persistent infection in MEFs. MEFs infected by SeV for 8 to 33 days were harvested, and total RNA extracted. The expression of viral NP and the IFNβ gene was analyzed by RT-PCR. **C**. SeV is present in all PI-MEFs. Control MEFs or PI-MEFs were fixed and subjected to immunofluorescent staining with an anti-SeV antibody (green). Blue: DAPI staining for nuclei. Scale bar, 20 µm. **D**. Negative regulation of IFNβ expression in PI-MEFs. Control MEFs or PI-MEFs were subjected to a new dose of SeV infection, double strand RNA (dsRNA, poly I:C) transfection or CHX (50 µg/ml) for 6 hrs, control MEFs were also treated with culture medium from PI-MEFs for 6 hrs, and PI-MEFs were treated with recombinant IFNβ protein (1000 U/ml) for the same length of time, total RNAs were prepared and the expression of IFNβ, SeV NP and β-Actin were analyzed by RT-PCR.

Monitoring infected MEFs revealed a different scenario: new infectious virus particles were continuously generated and released into the medium. This conclusion was supported by hemagglutination inhibition assays with the culture medium (Fig. S7). Surprisingly, IFNβ expression was low despite abundant virus present in these cells. An example is shown in Fig. 2B where MEFs infected with SeV from 8 days to over one month, continued to produce SeV nucleocapsid protein (NP) transcripts while the expression of IFNβ was extremely low. This is in contrast to the robust and transient expression of the IFNβ gene during the initial 24 hrs of infection (Fig. 2A). Cell death [61] during this extended virus infection was not observed, and we have maintained these cultures for over one year. SeV actively replicates in these cells during the entire time course. Thus, we have established a SeV persistent infection in MEFs; SeV and host cells coexist due to equilibrium between viral replication and host cell metabolism. An important feature of these cells is that the expression of the IFNβ gene is extremely low despite a high viral load. Based on this unexpected finding, we initiated studies of the regulation of IFNβ expression in these persistently infected MEFs (PI-MEFs). All of the PI-MEFs used in this study were cultured between 2–8 months after the initial SeV infection.

We carried out additional assays to confirm the presence of virus in the PI-MEFs. Immunofluorescent staining with an antibody specific for SeV particles revealed that all of the cells were positive for SeV in PI-MEFs, while no signal was detected in control MEFs (Fig. 2C). However, we noticed that the number of virus particles was highly variable between individual PI-MEFs. We also found that total RNA extracted from PI-MEFs was as potent an inducer of IFNβ gene expression as the RNA extracted directly from SeV viral stock when transfected into cells not previously exposed to infection (Fig. S8). This observation suggests that SeV pathogen-associated molecular patterns (PAMP) remain intact in PI-MEFs.

### IFNβ expression in PI-MEFs is inducible by CHX treatment

As an initial characterization of IFNβ regulation in PI-MEFs, we asked whether IFNβ gene expression could be reactivated by various inducers. We tried a new dose of SeV, or treatment with recombinant IFNβ protein, dsRNA (poly I:C), or the translation inhibitor CHX (Fig. 2D). Not surprisingly, infection of PI-MEFs with a new dose of SeV did not induce the expression of the IFNβ gene, considering that the cellular load of virus was already high. Similarly, treating PI-MEFs with recombinant IFNβ did not induce the expression of the IFNβ gene. dsRNA stimulation only weakly activated the expression of IFNβ in PI-MEFs, and the level was much reduced compared to control MEFs (Fig. 2D). However, a much larger stimulation of IFNβ expression was observed when the PI-MEFs were treated with CHX alone. By comparison, IFNβ expression was only weakly induced when control MEFs were treated with CHX (Fig. 2D). Consistent with the finding that viral particles are released from PI-MEFs, culture medium from these cells strongly induced the expression of IFNβ in control MEFs (Fig. 2D). Taken together, these data show that SeV actively replicates in PI-MEFs and strongly represses the expression of the IFNβ gene. The ability of PI-MEFs to produce IFNβ is greatly impaired when exposed to dsRNA. Strikingly, inhibiting protein synthesis by CHX activates the expression of IFNβ to a high level in PI-MEFs. It therefore appears that PI-MEFs produce viral or cellular proteins that block IFNβ gene expression, and CHX prevents the synthesis of these proteins. The response of PI-MEFs to selective stimulators provides the opportunity to identify key steps in the signaling pathway of IFNβ activation that is blocked by persistent infection.

### The block of IFNβ expression in PI-MEFs

As mentioned before, the signaling pathway leading to the activation of IFNβ expression by SeV infection is well established [6]. To identify the step(s) at which the block to IFNβ expression occurs in PI-MEFs, we over-expressed individual signaling components in the IFNβ activation pathway in both control MEFs and PI-MEFs. We then monitored the expression of the endogenous IFNβ gene in the absence or presence of a new SeV infection. A similar transfection efficiency was observed in control and PI-MEFs as indicated by comparable expression of GFP in both cell types (Fig. S9), thus excluding the possibility that any difference observed could be due to reduced transfection efficiency in PI-MEFs.

**Figure 3:**
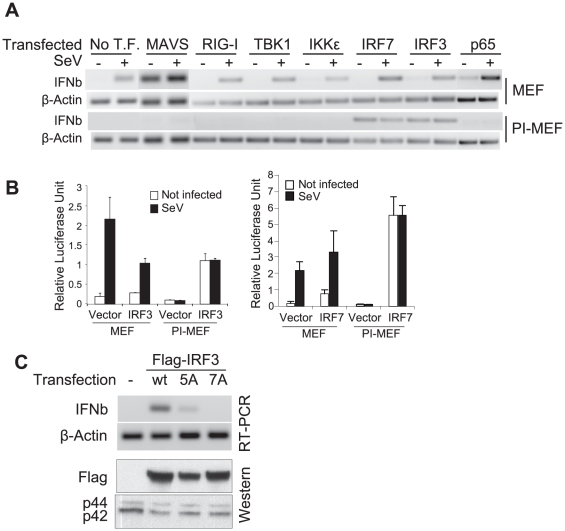
The block to IFNβ activation in PI-MEFs is at the level of IRF3/7. **A**. IRF3/7 transfection activates IFNβ gene expression in PI-MEFs. Control MEFs or PI-MEFs were transfected with plasmids encoding RIG-I, MAVS, TBK1, IKKε, IRF3, IRF7 and p65 genes. 24 hrs later, cells were infected with SeV for additional 6 hrs and RNA harvested for the analysis of IFNβ and β-Actin expression by RT-PCR. **B**. IRF3/7 activates an IFNβ gene reporter in PI-MEFs. Control MEFs or PI-MEFs were transfected with the IFNβ promoter firefly luciferase reporter gene together with IRF3 or IRF7 expression constructs. A renilla luciferase construct was also included for transfection reference. 24 hrs later, the cells were infected with SeV, and the luciferase activity measured one day later. **C**. IRF3 mutations rendering the protein defective for phosphorylation failed to activate IFNβ expression in PI-MEFs. PI-MEFs were transfected with a Flag-tagged wild type IRF3 expression construct or 5A and 7A mutants for 30 hrs. RNA and protein were harvested and analyzed for the expression of IFNβ and β-Actin by RT-PCR, or probed with anti-Flag and MAP kinase p42/p44 antibodies by western blot analysis.

As expected from previous studies [11], [12], [13], [14], over-expression of the MAVS protein strongly induced the expression of endogenous IFNβ in control MEFs, even in the absence of viral infection, and SeV-induced IFNβ expression was enhanced in cells in which MAVS was over-expressed (Fig. 3A). In contrast, expression of the IFNβ gene was only weakly induced in PI-MEFs by MAVS over-expression. This weak induction did not increase when a new dose of SeV infection was applied. The inability of MAVS to induce IFNβ expression in PI-MEFs was not due to reduced levels of MAVS, since western blot analysis showed similar if not higher levels of over-expressed MAVS in PI-MEFs compared to control cells (Fig. S10). We conclude that the block to IFNβ production in PI-MEFs is downstream from the MAVS protein in the IFNβ induction signaling pathway. Similarly, neither over-expression of RIG-I, or the IRF3/7 kinases TBK1 or IKKε was sufficient to activate IFNβ expression in PI-MEFs, suggesting that the block to IFN production lies downstream of these signaling components (Fig. 3A). By contrast, over-expression of the transcription factors IRF3 or IRF7 strongly activated IFNβ expression, independent of new SeV infection in PI-MEFs (Fig. 3A). By contrast, over-expression of the NFκB p65 subunit only weakly reactivated IFNβ expression in PI-MEFs, although it boosted SeV-induced IFNβ expression in control MEFs. Thus, it appears that a primary block to IFNβ expression in PI-MEFs is at the level of IRF3/7 proteins. Consistent with this possibility, we found that an IFNβ promoter driven luciferase reporter gene was strongly activated by IRF3 or IRF7 in PI-MEFs with or without SeV infection (Fig. 3B). The basal level of luciferase activity was very low in PI-MEFs in the absence of IRF3/7 transfection, indicating that the expression of the IFNβ reporter gene, like the endogenous gene, is blocked in PI-MEFs.

Phosphorylation of specific serine residues in the C-terminus IRF3 is required for IFNβ gene activation [52], [62]. To test whether exogenously expressed IRF3 can bypass this requirement, expression constructs for mutant IRF3 proteins 5A or 7A (5A: S396, S398, S402, S404 and S405 were all mutated to alanines, 7A has additional S385 and S386 mutated to alanine) [53] were transfected into PI-MEFs and induction of the endogenous IFNβ gene monitored. As expected, the 5A mutation only weakly activated, while the 7A mutant completely failed to activate the expression of the IFNβ gene in PI-MEFs (Fig. 3C). This experiment shows that phosphorylation of IRF3 is also required for the activation of IFNβ expression in PI-MEFs; and upstream kinases responsible for IRF3/7 phosphorylation can be activated (or already activated) in PI-MEFs. Moreover, the exogenous, but not the endogenous IRF3, is responsible for the activation of IFNβ expression in these over-expression assays. It is likely that endogenous IRF3 is inhibited in PI-MEFs, and this inhibition could not simply be relieved by over-expression of upstream components. However, this inhibition is sensitive to CHX treatment.

### IRF3 is negatively regulated in PI-MEFs

**Figure 4:**
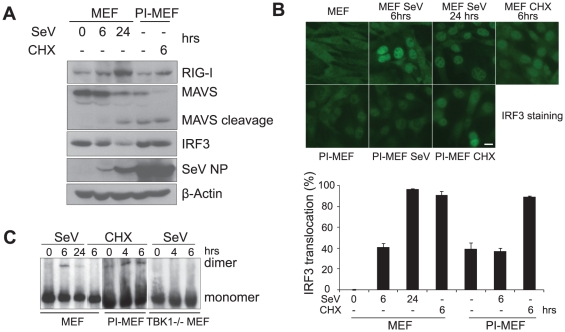
Regulation of IFNβ activation pathway signaling molecules in PI-MEFs. **A**. Expression of RIG-I, MAVS , IRF3, SeV NP and β-Actin in PI-MEFs. Control MEFs infected with SeV for the indicated times and PI-MEFs before and after CHX treatment were harvested; whole cell lysates were prepared and separated by SDS-PAGE, and western blots probed with antibodies specific for the indicated proteins. **B**. Subcellular localization of IRF3 in control MEFs and PI-MEFs infected with SeV, or treated with CHX. Cells were subjected to various treatments for indicated time, and fixed for IRF3 staining. The percentage of cells (out of ∼150 cells) with nuclear IRF3 staining under different conditions was determined and shown below the images. Data represent at least three independent experiments. Scale bar,10 µm. **C**. IRF3 dimerization in virus infected MEFs and CHX treated PI-MEFs. Control MEFs were infected with SeV, PI-MEFs were treated with CHX for indicated time. Total cell lysates were prepared and subjected to native gel electrophoresis and probed with IRF3 antibodies. TBK1 knockout MEFs were also infected with SeV in parallel for a negative control.

To further investigate the mechanism of IFNβ repression in PI-MEFs, we performed western blot analysis to monitor the expression of various signaling molecules in PI-MEFs compared to control MEFs. The abundant nucleocapsid protein (NP) from PI-MEFs confirmed the high load of virus in these cells (Fig. 4A). We found that the level of RIG-I protein in PI-MEFs returned to pre-induction levels in MEFs. However, MAVS remained cleaved and degraded in PI-MEFs as observed in control MEFs 24 hrs post acute SeV infection (Fig. 4A). This observation suggests that the mechanism responsible for MAVS cleavage and degradation is constitutively active in PI-MEFs. In addition, IRF3 protein levels were comparable or only slightly down in PI-MEFs compared to that of control MEFs. Since we have established that the major block to IFNβ expression is at the level of IRF3/7 protein (Fig. 3A), and IRF7 protein is too low to be detected in PI-MEFs, we focused our attention on IRF3 regulation PI-MEFs.

Virus infection induces IRF3 protein phosphorylation, homodimerization and nuclear translocation [63], [64]. These events can be visualized by immunofluorescent (IF) staining assays using an anti-IRF3 antibody. At 6 hrs post-SeV infection, a significant fraction of MEFs (∼40%) displayed strong IRF3 signals in the nucleus (Fig. 4B) and the number of cells in which this was the case increased over time despite the decrease in the total level of IRF3. At 24 hrs post-infection, IRF3 was observed in the nucleus of most cells despite a significantly reduced level of expression (Fig. 4B, and Fig. 1A). This observation, in conjunction with the time course of IRF3 degradation indicates that virus-induced degradation of IRF3 likely occurs in the nucleus. Immunofluorescent staining revealed that in contrast to the late time point of infection in control MEFs, where IRF3 was observed in the nucleus of greater than 95% cells, PI-MEFs display nuclear IRF3 signal in only about 40% of the cells. This fraction did not increase when the cells were subjected to a new SeV infection (Fig. 4B). The reduced fraction of cells in which IRF3 is in the nucleus in PI-MEFs compared to 24 hrs after initial infection suggests that some of the nuclear IRF3 was either degraded or exported during the establishment of persistent infection.

Notably, CHX treatment significantly increased the fraction of PI-MEFs with nuclear IRF3 signal to about 90% (Fig. 4B), and also increased the level of IRF3 dimers detected by native gel analysis (Fig. 4C). It is important to note that low levels of IRF3 dimers are constitutively present in PI-MEFs. CHX also induced the nuclear translocation of IRF3 in control MEFs (observed in >90% cells, Fig. 4B), but only weakly induced the expression of the IFNβ gene (Fig. 1A and 2D). In addition, the nuclear IRF3 in CHX treated MEFs was not detected as a dimer by native gel electrophoresis (Fig. 4C). It is therefore possible that the activation of IFNβ gene expression by CHX in PI-MEFs is primarily through the relief of the nuclear inhibition of IRF3 in these cells. However, it is also likely that the IRF3 protein activated by CHX acquired the ability to activate the IFNβ gene in the presence of SeV in PI-MEFs.

Taken together, these data suggest that in PI-MEFs there is cytoplasmic inhibition that prevents IRF3 activation (IRF3 remained in the cytoplasm, observed in ∼50–60% of the cells), and nuclear inhibition that suppress the transcriptional activity of IRF3 (observed in the remaining cells, with IRF3 stayed in the nucleus). The strong IFNβ induction by CHX is likely due to the relief of inhibition in both the nucleus and cytoplasm.

### Antiviral drug treatment activates IFNβ expression in PI-MEFs

**Figure 5:**
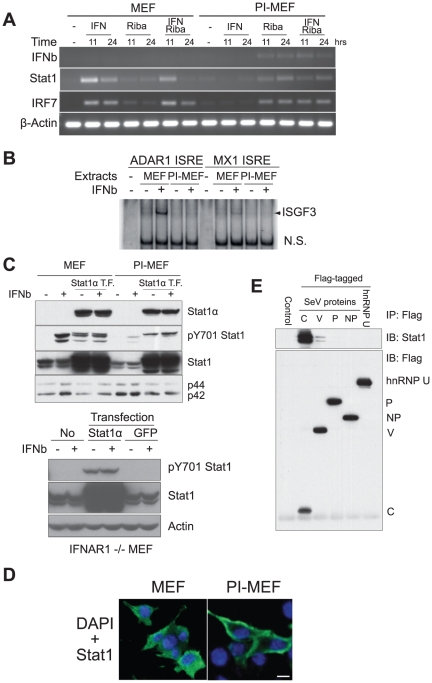
Interferon signaling is defective in PI-MEFs. **A**. ISGs are not activated by recombinant IFNβ in PI-MEFs, but ribavirin treatment activated the expression of ISGs and IFNβ. Control MEFs and PI-MEFs were treated with recombinant IFNβ (1000 U/ml) or ribavirin (100 µg/ml) either alone or in combination for 11 and 24 hrs. Cells were harvested for total RNA extraction, and the expression of IFNβ, Stat1, IRF7 and β-Actin mRNAs were analyzed by RT-PCR. **B**. ISGF3 complex formation is blocked in PI-MEFs. Control MEFs and PI-MEFs were stimulated with recombinant IFNβ protein for 1 hr, and total cell extracts prepared and assayed for the ISGF3 formation on ISRE DNA probes derived from the ADAR1 and MX1 genes. **C**. Reduced phosphorylation of STAT1 tyrosine 701 in PI-MEFs after IFN treatment. Top: Control MEFs and PI-MEFs were transfected with a plasmid encoding Stat1α protein for 24 hrs, transfected and non-transfected cells were stimulated with recombinant IFNβ for 1 hr. Total protein was prepared for western blot analysis with antibodies against Stat1, phospho-tyrosine 701 Stat1, Stat1α and MAP kinase p42/p44. Bottom: the same experiments were conducted in IFNAR1 deficient MEFs and the expression of Stat1 and phospho-Y701 Stat1 were analyzed by western blotting. GFP was also transfected as a control. **D**. Plasma membrane localization of Stat1 protein in PI-MEFs. Control MEFs and PI-MEFs were transfected with a plasmid encoding Flag-tagged Stat1 protein. 24 hrs later cells were fixed and subjected to immunofluorescent staining with an anti-Flag antibody (green). Blue: DAPI staining for nuclei. Scale bar, 10 µm. **E**. SeV C protein specifically interacts with Stat1 protein. Constructs for Flag-tagged SeV C, V, P and NP proteins were transfected into MEFs, 24 hrs later, whole cell extracts were prepared and subjected to anti-Flag M2 bead immunoprecipitation (IP). The associated proteins were eluted and separated on SDS-PAGE, blotted and probed with an anti-Stat1 antibody. An hnrnp U expression construct was also included as a control. IB: immunoblot.

The inhibition of IFNβ gene expression in PI-MEFs is similar to that observed with persistent viral infections where the expression of IFN genes is suppressed [45]. Thus, PI-MEFs could serve as a model system to test the effects of anti-viral drugs. While we found that treating these cells with MG132 did not induce the expression of IFNβ, we found ribavirin, an anti-RNA virus drug [65], induced the expression of the IFNβ gene in PI-MEFs. As shown in Fig. 5A, ribavirin treatment of PI-MEFs induced the expression of IFNβ, IRF7 and STAT1, in contrast to the weak induction of IRF7 and STAT1 but not IFNβ in control MEFs (Fig. 5A). We note from immunofluorescent staining experiments that the activation of the IFNβ gene by ribavirin in PI-MEFs is not due to increased IRF3 nuclear localization. Sustained treatment of PI-MEFs with ribavirin significantly reduced the virus load in these cells, and partially restored the induction of IFNβ by a new dose of virus infection (Fig. S11).

Since ribavirin and IFN are frequently used in combination to treat chronic HCV infections [65], we also tested whether recombinant IFNβ can enhance the induction of the IFNβ gene by ribavirin in PI-MEFs. The effects were found to be minimal (Fig. 5A). Surprisingly, treating PI-MEFs with recombinant IFNβ failed to induce the expression of IRF7 and STAT1, although they were strongly induced by IFNβ in control MEFs (Fig. 5A). Thus, it appears that not only is the IFNβ activation pathway blocked in PI-MEFs, but the IFN signaling pathway is blocked as well.

### Interferon signaling is blocked in PI-MEFs

The observation that recombinant IFNβ did not induce IRF7 and STAT1 expression in PI-MEFs suggests that IFN signaling is blocked in these cells. To test this possibility, we examined the hallmark of IFN signaling: formation of the ISGF3 complex and phosphorylation of STAT1 upon IFN stimulation [66]. In control cells, IFN treatment induced the formation of the ISGF3 complex on an ADAR1 promoter probe, while this complex was virtually absent in PI-MEFs treated with IFN (Fig. 5B). Although the binding was weaker, the MX1 promoter probe revealed the same pattern (Fig. 5B). These observations show that PI-MEFs are defective in the induction of ISGF3 complex formation by IFN.

The failure to detect inducible ISGF3 binding could be due to either reduced expression of the ISGF3 components (STAT1, STAT2 and IRF9), reduced accessibility of STAT protein to JAK/TYK kinase, or reduced kinase activity in these cells. We therefore carried out a western blot analysis and probed the blot for total and tyrosine (Y) 701 phosphorylated STAT1. The basal level of STAT1 protein in PI-MEFs was 2–3 fold lower than that in control MEFs (Fig. 5C). In addition, the phospho-Y701 STAT1 level was much lower (>5 fold) than that in IFN-stimulated control cells (Fig. 5C). Thus, in addition to the reduced levels of STAT1 protein in PI-MEFs, it appears that the Jak/STAT pathway is also inhibited, accounting for the reduced Y701 phosphorylation.

We asked whether the kinases responsible for Y701 phosphorylation were impaired in PI-MEFs. To compensate for the reduced level of STAT1 in PI-MEFs, we exogenously expressed Flag-tagged STAT1α protein in control MEFs and PI-MEFs, and monitored its Y701 phosphorylation after IFN stimulation. Unexpectedly, STAT1 over-expression was sufficient to induce similar levels of Y701 phosphorylation of the exogenous STAT1 in both control MEFs and PI-MEFs (it also induced Y701 phosphorylation of the endogenous STAT1 protein in control MEFs, Fig. 5C, top panel). IFN stimulation only weakly increased the Y701 phosphorylation level in both control MEFs and PI-MEFs. Since STAT1 over-expression did not induce IFNβ expression in PI-MEFs (Fig. S12), the Y701 phosphorylation observed in these cells suggests that STAT1 can be phosphorylated independent of IFN signaling. This was indeed confirmed by experiments with IFN receptor deficient (IFNAR1 −/−) MEFs, where over-expression of STAT1 induced Y701 phosphorylation (Fig. 5C, bottom panel). Taken together, these data suggest that kinase activity appears to be intact in PI-MEFs, but the reduced expression of STAT1 and other mechanisms resulted in reduced phosphorylation after IFN stimulation.

To explore additional mechanisms regulating STAT1 in PI-MEFs, we monitored its intracellular distribution by transfecting Flag-tagged STAT1 into cells followed by immunofluorescent staining of the Flag peptide. In transfected control MEFs, the cytoplasmic STAT1 was evenly distributed (Fig. 5D). However, in PI-MEFs STAT1 was highly enriched in plasma membrane fractions (observed in ∼35% cells) (Fig. 5D). The aberrant localization of STAT1 protein in PI-MEFs is likely to also contribute to the defects of IFN signaling in these cells.

The SeV C protein has been reported to specifically interact with STAT1, and lead to its degradation [67], [68]. Recently it was also shown that the C protein can localize to the plasma membrane [69]. Consistent with these observations, we were able to use Flag-tagged C protein to pull down endogenous STAT1 from MEFs (Fig. 5E). Therefore, it is likely that the block to IFN signaling and the aberrant localization of STAT1 are caused, at least in part, by the viral C protein in PI-MEFs.

### Viral genes required for the establishment of persistent infection

Both the translation inhibitor CHX and the antiviral drug ribavirin activated IFNβ gene expression in PI-MEFs, suggesting that viral protein(s) might be directly involved in the repression of IFNβ expression. As a member of the paramyxovirus family, SeV encodes 6 major open reading frames (ORFs), corresponding to nucleocapsid (NP), phosphorylation (P), matrix protein (M), fusion protein (F), hemagglutinin neuraminidase (HN) and large (L) protein along the sense strand of its RNA genome. The phosphorylation (P) ORF can also give rise to a small protein C (mentioned above, with a different starting site and a different frame of ORF) and another protein V (shares a common N-terminus with the P protein, but has a unique cysteine rich C-terminus) [46]. We generated expression constructs bearing cDNAs encoding each of these proteins, and tested whether any of them inhibit the induction of IFNβ by various inducers.

**Figure 6:**
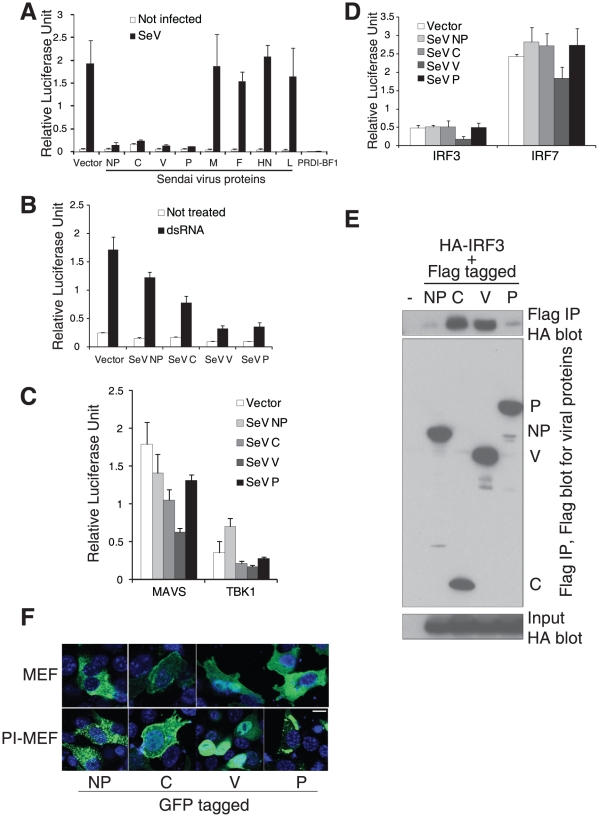
SeV encoded proteins inhibit IFNβ expression. **A**. NP, C, V and P proteins inhibit IFNβ reporter induction by SeV. Expression constructs for SeV proteins were cotransfected into 293T cells with an IFNβ promoter driving firefly luciferase expression plasmid together with a renilla luciferase reporter. 24 hrs later the cells were infected with SeV and luciferase activities measured after an additional 24 hrs.The PRDI-BF1 expression construct was also transfected as a control. **B**. SeV C, V and P proteins suppress IFNβ induction by dsRNA (poly I:C). Experiments were conducted as in A, but dsRNA (2 µg/ml) was used instead of SeV to induce IFNβ expression. **C**. MAVS and TBK1 induced IFNβ expression is strongly suppressed by the SeV V protein. The IFNβ promoter driving firefly luciferase reporter was transfected with a reference renilla construct together with MAVS or TBK1 expression constructs. SeV NP, C, V and P protein expression constructs were also included separately, luciferase activities were measured 24 hrs later. **D**. The V protein of SeV inhibits IFNβ induction by the over-expression of IRF3 or IRF7 protein. Experiments were conducted as in C, but IRF3 and IRF7 expression constructs were cotransfected instead of MAVS or TBK1, luciferase activities were measured 24 hrs later. **E**. Interactions between SeV protein and IRF3. Plasmids encoding Flag-tagged SeV NP, C, V and P proteins were cotransfected with plasmid encoding HA-tagged IRF3, total cell lysates were prepared 24 hrs later and subjected to immunoprecipitation with anti-Flag beads. The associated proteins were separated in SDS-PAGE and blot with anti-HA or anti-Flag antibodies. **F**. Distinct localization of SeV NP, C, V and P proteins. GFP-fusion constructs for NP, C, V and P proteins of SeV were generated and transfected into control MEFs and PI-MEFs. 24 hrs after transfection, cells were fixed and directly analyzed with confocal microscopy. Blue: DAPI staining for nuclei. Scale bar, 10 µm.

Luciferase reporter assays showed that four out of the eight proteins tested, NP, C, V and P, strongly inhibited the induction of the IFNβ reporter when SeV was used as the inducer (Fig. 6A). V and P proteins also significantly attenuated IFNβ induction by dsRNA in reporter assays (Fig. 6B). To identify the specific signaling components affected by these proteins, we conducted luciferase reporter assays with over-expression of MAVS, TBK1, IRF3 and IRF7 proteins. Although each viral protein showed considerable inhibition of IFNβ induction when MAVS was over-expressed, the inhibition by V protein was the strongest (Fig. 6C). Significant inhibition of the IFNβ reporter by the V protein was also observed with TBK1 over-expression (Fig. 6C). Unexpectedly, instead of the inhibition seen in MAVS induction, NP expression stimulated IFNβ induction by TBK1 (Fig. 6C). We note that the V protein is the only viral protein tested, that decreases the induction of the IFNβ reporter when co-expressed with IRF3 or IRF7 (Fig. 6D).

To determine whether the V protein can interact with IRF3, we transfected 293T cells with Flag-tagged NP, C, V and P proteins together with HA tagged IRF3 protein. Viral proteins were immunoprecipitated using anti-Flag beads, and separated by SDS-PAGE. A Western blot using anti-HA antibodies as a probe revealed that the V protein strongly interacts with IRF3 (Fig. 6E). The same experiment showed that the C protein also interacts with IRF3, although it did not inhibit the transactivation activity of IRF3 in the IFNβ reporter assays (Fig. 6D, 6E).

We also examined the localization of these viral proteins in MEFs by expressing GFP fusion constructs. Consistent with a previous report [69], we observed that C protein localized to the plasma membrane, but was also detected in the cytoplasm and nucleus (Fig. 6F). The V protein is detected in both the cytoplasm and nucleus in both control and PI-MEFs (Fig. 6F). Since the life cycle of SeV is exclusively in the cytoplasm (structural components of SeV are all made in the cytoplasm), the detection of C and V proteins in the nucleus highlights their functions in antagonizing IFN activation and signaling. Strikingly, while the NP and P proteins were distributed evenly in the cytoplasm in control MEFs, signals of these two proteins displayed aggregated patterns in PI-MEFs. It is likely that these transfected proteins were recruited to the sites of virus assembly in PI-MEFs.

### shRNA targeting SeV PVC gene dramatically changes SeV-induced cellular innate immune response

**Figure 7:**
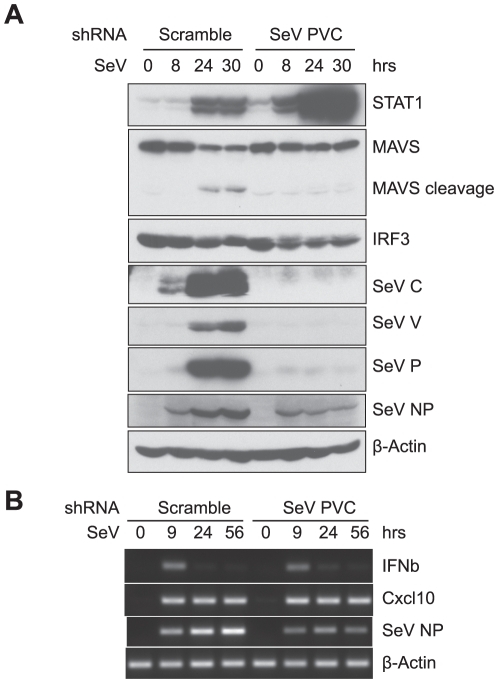
Interfering SeV replication with shRNA affects innate immune response. **A**. Targeting SeV by shRNA modulates the expression of critical factors in innate immunity. MEFs were treated with shRNA specifically targeting the SeV PVC gene or a scramble sequence shRNA as control. Cells were then infected with SeV. Total protein lysates were prepared after indicated time and separated on SDS-PAGE for western blot analyses with anti-STAT1, MAVS, IRF3, SeV C, V, P and NP and β-Actin antibodies. **B**. Targeting SeV by shRNA has minimal effects on IFNβ expression kinetics. Experiments were conducted similarly as in A, but cells were harvested after indicated time for RNA extraction instead. The expression of IFNβ, Cxcl10, SeV NP and β-Actin was analyzed by RT-PCR.

To directly test the effects of inhibiting SeV replication on IFNβ expression in PI-MEFs, we knocked-down the expression of the SeV encoded genes by shRNA. However the knockdown efficiency was not satisfactory probably due to the high abundances of the viral loads in these cells. We thus treated MEFs with shRNA specifically targeting SeV PVC gene and then infected with SeV for increasing times. Western blot analyses revealed that the production of SeV C, V and P proteins was abolished by this shRNA treatment, and the increasing amounts of the NP protein overtime in the infected control cells treated with a scramble shRNA was not observed (Fig. 7A). Notably, the degradation of IRF3 was similarly observed in both cells (Fig. 7A), correlating the similar IFNβ turn-off kinetics in cells treated with both shRNAs (Fig. 7B). This observation suggests that the degradation of IRF3 is most likely the result of host cell proteins. Strikingly, the induction of STAT1 was dramatically enhanced in cells treated with PVC shRNA, and the cleavage and degradation of MAVS was also reduced in these cells. These data clearly show that SeV encoded proteins are indeed capable of antagonizing cellular innate immunity by targeting critical signaling molecules.

## Discussion

### Post-induction IFNβ turn-off

Here we show that inactivation of the transcription factor IRF3 plays a critical role in the negative regulation of IFNβ expression during both acute and persistent Sendai virus infections. Western blot analyses reveal that the level of IRF3 correlates with IFNβ gene expression during acute virus infection. The degradation of IRF3 leads to the termination of IFNβ transcription, and stabilization of IRF3 with CHX sustains IFNβ gene transcription. Turnover of IRF3 would prevent enhanceosome assembly [70], and thereby turn-off IFNβ expression. Activation of IFNβ gene expression requires IRF3, IRF7 and NFκB [70], but we find that the levels of IRF7 and p65 remain relatively constant during the course of viral infection (Fig. 1A). This observation correlates with the essential role of IRF3 in the activation of the IFNβ gene, as deletion of IRF3 in mice leads to a dramatic reduction in the level of IFNβ expression after virus infection [71]. IRF7 is required for high levels of IFN gene expression [72], but it is present at only low levels prior to virus infection [72]. Moreover, the super induction of IFNβ by CHX treatment of virus infected cells [37], which leads to high levels of IFNβ mRNA, occurs in the absence of IRF7 induction. Thus, it appears that IRF3 plays a unique and essential role in IFNβ induction.

While we were able to confirm previous studies showing that proteosome inhibition by MG132 treatment stabilizes IRF3 [52], [53], we show that the proteosome is not the sole mechanism for IRF3 degradation. Specifically, we find that significant IRF3 degradation can be observed in virus infected cells treated with MG132 for 24 hrs. In fact, CHX appears to be a more efficient inhibitor of the virus-induced degradation of IRF3. While it is possible that CHX treatment prevents the synthesis of an inducible E3 ligase that targets IRF3 for degradation, we propose that one or more proteosome-independent proteases that target IRF3 are induced by virus, leading to the termination of IFNβ transcription (a model of IRF3 inactivation during acute virus infection is shown in Fig. S13).

Induced IRF3 degradation was previously observed with other virus infections. For example, the paramyxovirus family Newcastle disease virus and Measles virus induce IRF3 degradation in various cell lines [73], [74]. Moreover, vesicular stomatitis virus also induces IRF3 degradation in A549 cells [75], and dsRNA (poly I:C) induces strong turnover of IRF3 in MEFs [53], [75]. Additional studies will be required to determine whether the loss of IRF3 in these cases correlates with the kinetics of IFNβ down regulation. Assuming that this is the case, IRF3 degradation may serve as a general mechanism for turning off IFNβ gene expression.

We also present evidence that the two repressor proteins PRDI-BF1 and PRDII-BF1, which bind specifically to the IFNβ enhancer, and were thought to be involved in post-induction IFNβ repression [36], [55], do not appear to be required in knock-out mouse cells. We find that MEFs deficient for either PRDI-BF1 or PRDII-BF1 display the same kinetics of IFNβ turn-off as wild type cells after virus infection (Fig. S3). The possibility that the two repressors are redundant was ruled out by the observation that shRNA knockdown of PRDII-BF1 in PRDI-BF1 knockout MEFs display normal kinetics of IFNβ down regulation after virus infection (Fig. S4C). Although we cannot rule out the possibility that one or both of these proteins function as post-induction repressors of IFNβ gene expression in other cells, it would appear that IRF3 turnover is the primary mechanism of post-induction repression.

### Negative regulation of the IFNβ gene in persistent infected cells

We have shown that SeV infection of MEFs can lead to a persistent infection in which IFNβ gene expression is repressed and viral replication continues. As is the case *in vivo*, where different tissues display varying susceptibilities to virus infection [76], we find that the ability to establish persistent infection depends on the cell type. For example, we show that SeV kills L929, Raw264.7, MG63 and Hela cells, while the virus is cleared from Namalwa cells. In the case of MEFs, viral replication must establish equilibrium with cellular metabolism, leading to long-term cell survival. An important feature of persistently infected MEFs is that IFNβ production is strongly repressed even though the virus continues active replication. Examination of the expression of signaling components in PI-MEFs, and of late time points during acute SeV infections revealed that the MAVS protein is cleaved and degraded in both cases. We have mapped the cleavage sites on MAVS (Fig. S6), but the mechanism and function of MAVS cleavage and degradation remain to be investigated.

We have identified two non-redundant inhibitory mechanisms that operate in PI-MEFs to prevent IFNβ activation. One acts in the cytoplasm to inhibit the activation of IRF3 and its nuclear translocation. The second acts in the nucleus, to prevent IFNβ gene activation by the nuclear IRF3 protein. We show that IRF3 is activated and migrates to the nucleus in a significant fraction (∼40%) of PI-MEFs (Fig. 4B), indicating that the upstream signaling pathway is functioning. This observation also suggests that the cleavage and degradation of MAVS might be a byproduct of signal activation, rather than a critical step (i.e. termination of the activated signaling cascade). The transcriptional activity of nuclear IRF3 in PI-MEFs appears to be blocked, and this block can be overcome by expressing exogenous IRF3 or IRF7 (Fig. 3A). We speculate that the excess IRF3 or IRF7 in these experiments blocks the negative factors by competitive inhibition. CHX or ribavirin treatment also activates endogenous IFNβ expression in PI-MEFs (Fig. 2D, 5A), suggesting that viral proteins are directly involved in these inhibitory mechanisms.

We find that the SeV NP, C, V and P proteins can all inhibit the induction of IFNβ by SeV (Fig. 6A). It is possible that NP and P proteins interfere with the detection of the viral RNA by cellular sensors, as both proteins associate with the viral RNA genome as structural or polymerase components [46]. Notably, the nucleoproteins from many Arenaviruses can also inhibit type I IFN expression [77], [78], it is likely they also interfere with the virus detection step.

In contrast to some V proteins from Rubulavirus, which inhibit IRF3 activation by competing with the TBK1/IKKε kinases [79], we find that the V protein from SeV directly inhibits the activity of IRF3, thus providing a clear mechanism for the suppression of IFNβ expression in PI-MEFs. This is consistent with a previous observation that SeV V protein inhibits IFNβ activation [80]. In addition to published results that SeV V protein can specifically inhibit the activity of the RNA sensor MDA-5 [81], [82], we found that the V protein potently inhibits the induction of an IFNβ reporter by virus, dsRNA and over-expression of MAVS or TBK1 (Fig. 6A–C). Importantly, the V protein is the only viral protein capable of inhibiting IFNβ induction by IRF3/7 over-expression in reporter assays (Fig. 6D). The physical interaction between the V protein and IRF3 was demonstrated by co-immunoprecipitation experiments (Fig. 6E). The V protein is found in both the cytoplasm and nucleus (Fig. 6F); and therefore, has the potential to inhibit IRF3 activity in both compartments. The finding that the V protein specifically targets IRF3 is also supported by an *in vivo* study, where recombinant SeV devoid of the V protein was rapidly cleared from infected mice, except ones deficient for IRF3 gene [83]. We propose that the inhibition of IRF3 by the V protein is the primary mechanism for the repression of IFNβ expression in persistently infected cells (Fig. S13).

The mechanism by which the C protein inhibits IFNβ induction is less clear. The C protein can also interact with IRF3 in over-expression experiments, but the inhibition appears to occur upstream of IRF3/7. The C protein inhibited the induction of the IFNβ reporter by both MAVS and TBK1, but not IRF3/7 (Fig. 6D). In previous studies the C protein was shown to antagonize IFN signaling by specifically interacting with STAT1 and interfering with its activity (Fig. 5E, [67]). Similarly, the C protein is also essential for the *in vivo* pathogenicity of SeV [84]. Thus, in our PI-MEFs, SeV suppresses the activation of the IFNβ gene, and also inhibits IFN signaling by targeting the key transcription factors IRF3 and STAT1. Both of these activities are required for persistent infection by SeV, as recombinant virus devoid of either C or V proteins is rapidly cleared from infected mice [83], [84]. However, the question of whether this is a general strategy used by other viruses to establish persistent infection remains to be answered.

## Materials and Methods

### Cells, chemicals, reagents and plasmids

Immortalized wild type MEFs were from Wen-chai Yeh (Toronto, Canada), 293T, Hela, MG63, Namalwa, L929, Raw264.7 cells are all from ATCC. Cycloheximide is from calbiochem, ribavirin, recombinant interferon-β and MG132 are from Sigma. Expression constructs for MAVS, TBK1, IRF3, IRF7 and Stat1α were described before [53], [85], [86], [87]. Flag-tagged expression constructs for SeV NP, C, V and P proteins were generated by cloning viral cDNAs to pcDNA3-Flag (Invitrogen) or pFlag-CMV2 (Sigma) vectors. GFP-fusion constructs for these proteins were generated by Gateway cloning with pcDNA-DEST53 (Invitrogen). Sendai virus cantell strain is purchased from Charles River laboratory. For viral infections, SeV was added directly into the medium at a concentration of 200–300 HAU/ml and incubated for indicated time. Procedures for lentivirus-mediated shRNA knockdown experiments were described before [86], sequences 5′-GAAGACCAAGCTGAAGGACTT-3′ and 5′-CGCTCAGAGGTGCAAGCTTAA-3′ were cloned to pLKO.1 vector to target SeV PVC and mouse Itch gene respectively.

### Luciferase assays

293T Cells or MEFs were transfected with IFNβ promoter driving firefly luciferase construct together with renilla luciferase construct as reference. Cells were either directly treated with SeV or double strand RNA (poly I:C), or co-transfected with other expression constructs. 24 hrs later cells were lysed and subjected to Dual-Glo luciferase assay analysis (Promega) with an Analyst AD plate reader.

### Antibodies, western blot and immunoprecipitation

Antibodies against RIG-I, rodent specific MAVS, TBK1, IKBα, MAP kinase p42/p44 are from Cell Signaling, anti-mouse IRF3, IRF7 antibodies are from Invitrogen. Anti-p65, human IRF3, Stat1, Stat1α antibodies are from Santa Cruz. β-actin, phosphor-Y701 Stat1 antibodies are from Abcam. Anti-Flag antibody and agarose beads are from Sigma. Anti-mouse MAVS antibody is a gift from Dr. James Chen (UT southwestern, Dallas TX). Anti-SeV antibody used for cell staining is a gift from Dr. Ben tenOever (Mount Sinai, New York). Anti-SeV NP serum is from Dr. Valery Grdzelishvili (Charlotte, USA). Anti-SeV C protein antibody is from Dr. Ganes Sen (Cleveland, USA). Anti-SeV P and V serum is from Dr. Atsushi Kato (National Institute of Infectious Diseases, Japan). Western blots were carried out according to standard protocols. About 50 µg of total protein lysate (lysed in a buffer of 20 mM Tris-HCl, pH7.5, 150 mM NaCl, 1% Triton X-100, 1 mM EDTA, 30 mM NaF, 1 mM glycerolphosphate, 1× proteinase inhibitor (Roche) and 1 mM Na_3_VO4) was denatured in sampling buffer (50 mM Tris-HCl, pH 6.8, 10% glycerol, 2% SDS, 0.02% bromophenol blue and 2.5% β-mecaptoethanol) and subjected to SDS-PAGE. Proteins were transferred to a PVDF membrane, blocked with 5% milk in Tris-buffered saline Tween 20 (TBST), and incubated with various primary antibodies solutions. Washed membranes were incubated with HRP conjugated secondary antibody and protein bands visualized with ECL reagents (Millipore or Pierce). Immunoprecipitation experiments were carried out by incubating anti-Flag M2 beads with about 500 µg of total protein lysates (prepared with the lysis buffer described above) for 2 hrs at 4°C, beads were collected and washed 4 times with the same buffer. Bound proteins were eluted in sampling buffer and subjected to western blot analysis.

### RNase protection assays, RT-PCR

RNase protection assays were conducted as described before [37], anti-sense probe for human IFNβ and gamma-actin were generated by *in vitro* transcription and gel purified. About 30 µg of total RNA were hybridized with probes over night at 50°C in a buffer (40 mM PIPEs, pH 6.8, 1 mM EDTA,0.4 M NaCl and 80% formamide) and RNAs not annealed were digested with RNase A/T1 mixture (Ambion). Samples were then denatured and separated in a denaturing gel. Dried gel was exposed to Phosphoimager. RT-PCR was conducted according to routine protocols, cDNAs were made from about 5 µg of total RNA by oligo-dT primer with AMV reverse transcriptase (Promega). Gene specific primers (Table S1) were used to amplify the desired products.

### Immunofluorescent staining

Immunofluoresence staining was conducted according to standard procedures. Cells were fixed with 4% formaldehyde in PBS for 10 min, and washed for 3 times, 5 min each. Cells were permeabilized with 0.1% Triton X-100 in PBS for 10 min, then washed again three times 5 min each. Primary antibody incubation was carried at 4°C over night. Cells were extensively washed, and incubated with FITC-conjugated 2^nd^ antibody. Slides were mounted and subjected to microscopy analysis. In the case of GFP fusion viral protein, transfected cells were fixed, washed and directly mounted before microscopy analysis.

### Electrophoresis mobility shift assays

About 10 µg of total cell lysates were incubated with 25 ng of radio-labeled ADAR1 or MX1 probe at 37°C for 20 min in a buffer of 12 mM Tris-HCl, pH8.0, 60 mM KCl, 2 mM MgCl2, 0.12 mM EDTA, 0.3 mM DTT and 6% glycerol, and resolved in a 5% native PAGE gel. Dried gels were exposed to Phosphoimager.

## Supporting Information

Figure S1 **Effects of proteosome inhibitors on virus-induced IRF3 degradation.** **A.** MG132 potently blocks TNFα induced IKBα degradation. MEFs were treated with recombinant TNFα (10 ng/ml) for 20 min in the presence or absence of MG132 (50 µM). Total protein lysates were prepared and subjected to western blot analysis for the detection of IKBα and β-Actin. **B.** Lactacystin does not completely block SeV-induced IRF3 degradation. MEFs were infected with SeV in the presence or absence of lactacystin (10 µM, a concentration known to potently inhibit proteosome activities [53], [88]) for 6 and 24 hrs. Total protein lysates were prepared and subjected to western blot analysis for the detection of IRF3 and β-Actin.
(EPS)

Figure S2 **Knocking down the expression of PRDI-BF1 in MG63 cells did not sustain IFNβ expression.** MG63 cells were transfected with siRNAs targeting PRDI-BF1 gene. 36 hrs after transfection, cells were infected with SeV for 8 hrs and 24 hrs, total RNA extracted for the analysis of IFNβ and GAPDH expression (top panel). The efficiency of knocking-down was monitored by western blot (bottom panel).
(EPS)

Figure S3 **Neither PRDI-BF1 nor PRDII-BF1 is required for IFNβ turn-off in MEFs.** **A**. PRDI-BF1 is not required for IFNβ turn-off after SeV infection. The diagram of PRDI-BF1 locus and the genotyping results of PRDI-BF1 deficient MEFs and control wild type cells are shown in the top panel, exons 6–8 are targeted for the deletion in the knockout cells. The kinetics of IFNβ expression in wild type and PRDI-BF1 deficient cells after SeV infection monitored by RT-PCR is shown in the bottom panel. **B**. PRDII-BF1 is not required for IFNβ turn-off. The PRDII-BF1 gene locus, genotyping results and IFNβ expression kinetics are shown the same as in A. The entire exon 3 (5939 bp) of PRFII-BF1 gene is deleted in the deficient cells.
(EPS)

Figure S4 **Knocking-down PRDII-BF1 expression in PRDI-BF1 knockout MEFs does not affect IFNβ turn-off.** **A**. Genotyping results of wild type MEF and PRDI-BF1 knockout MEFs treated with shRNA targeting PRDII-BF1 or a control scramble shRNA. Primer pair a in Fig. S3A was used for PRDI-BF1 detection, and ATP1a1 gene was amplified as a control. **B**. shRNA knockdown efficiency of PRDII-BF1. Scramble or PRDII-BF1 shRNA treated PRDI-BF1 knockout MEFs were left uninfected, or infected with SeV for 4 hrs, and total cellular RNA extracted for RT-PCR analysis of PRDII-BF1 and β-Actin expression. **C**. Normal post-induction turn-off of IFNβ in PRDI-BF1 knockout MEFs with reduced PRDII-BF1 expression. Scramble or PRDII-BF1 shRNA treated PRDI-BF1 knockout MEFs were infected with SeV for indicated time, cells were harvested and RNA extracted for the analysis of IFNβ and β-Actin expression by RT-PCR.
(EPS)

Figure S5 **Itch is not involved in the regulation of MAVS cleavage or degradation.** **A.** SeV induces degradation and cleavage of MAVS protein in Raw264.7 cells. Raw264.7 cells were infected with SeV for the indicated times, total protein lysates were prepared and analyzed for the expression of the MAVS protein by western blot. **B.** Knocking-down the expression of Itch does not affect SeV-induced MAVS cleavage and degradation. Wild type MEFs were treated with shRNA targeting Itch or a control scramble shRNA, and subjected to SeV infection. Total protein lysates were prepared after indicated time and subject to western blot for the expression of MAVS, Itch and β-Actin.
(EPS)

Figure S6 **Cleavage of MAVS is not required for IFNβ turn-off.** MAVS deficient MEFs were reconstituted (RC) with wild type or I441A mutant MAVS proteins, and subjected to SeV infection. Half of the samples were harvested for RT-PCR analysis of IFNβ and β-Actin expression (bottom panel), and the other half were analyzed by western blot for the cleavage and degradation of the MAVS protein (top panel).
(EPS)

Figure S7 **Culture medium of PI-MEFs contains virus particles.** Hemagglutination inhibition assays were conducted to confirm the release of virus particles into the culture medium of PI-MEFs. Series dilutions of culture medium from control MEFs or PI-MEFs (2 months after initial infection) or SeV stock were added to wells containing chicken red blood cells. Presence of virus inhibited the agglutination of these cells.
(EPS)

Figure S8 **Total RNA extracted from PI-MEFs is a potent IFNβ inducer.** Total RNA extracted from control MEFs, PI-MEFs or Sendai virus stock were transfected into control MEFs (8 µg of total RNA from control MEFs and PI-MEFs, and about 1 µg of SeV RNA were transfected into 2 million cells of control MEFs), 6 hrs later, total RNA were extracted and the expression of IFNβ and β-actin was analyzed by RT-PCR.
(EPS)

Figure S9 **Similar transfection efficiency of control MEF and PI-MEF.** A GFP expression plasmid was transfected into control MEFs and PI-MEFs, 24 hrs later, the expression of GFP was monitored by epifluorescent microscopy. Scale bar, 50 µm.
(EPS)

Figure S10 **Expression of HA-tagged MAVS in control MEFs and PI-MEFs.** About 2 million control MEFs and PI-MEFs were transfected with 8 µg of HA-MAVS expression construct, cells were lysed after 24 hrs and the expression of HA-MAVS was monitored by western blot with an anti-HA antibody. A non-specific (N.S.) band serves as the loading control.
(EPS)

Figure S11 **Treating PI-MEFs with ribavirin partially restores the induction of IFNβ expression.** Control PI-MEFs and PI-MEFs pretreated with ribavirin (25 µg/ml) for a week were infected with a new dose of SeV for 6 hrs. Total cellular RNA was extracted, and the expression of IFNβ, SeV NP and β-Actin was analyzed by RT-PCR.
(EPS)

Figure S12 **Over-expression of Stat1 did not induce IFNβ in PI-MEFs.** Control MEFs and PI-MEFs were transfected with an expression construct for Stat1. 24 hrs later, total RNA were extracted for RT-PCR analysis of IFNβ, IRF7 and β-Actin expression.
(EPS)

Figure S13 **Diagram showing proposed model of IRF3 inactivation during acute and persistent Sendai virus infections.** **Left:** During acute virus infection, the primary mechanism of IRF3 inactivation is proteolytic degradation, which leads directly to the post-induction turn-off of IFNβ expression. Although the ubiquitin-proteosome pathway plays an important role in IRF3 degradation, other unknown but inducible protease(s) (or factors involved in the activation of these proteases) also contribute significantly to IRF3 degradation. In fact, CHX has a larger effect on IRF3 levels than proteosome inhibitors, suggesting that the E3 ligase targeting IRF3 for degradation may also be inducible. Alternatively, it is possible that phosphorylated IRF3 can be inactivated by an inducible phosphatase and then exported to the cytoplasm. Whether IRF3 de-phosphorylation is required for its degradation is not clear. **Right:** During persistent SeV infection, the viral V protein directly inhibits IRF3 activity. Binding of the V protein to IRF3 could interfere with its DNA binding activity (as diagramed), or block its interaction with other co-activators (not diagramed), thus inhibiting IFNβ gene activation. Our data do not exclude the possibility that a labile host factor (labile factor X) also inhibits IRF3 activity in persistently infected cells. Both the V protein and the putative labile factor X inactivate IRF3 by inhibiting its transcriptional activity, not necessarily leading to degradation.
(EPS)

Table S1 **Sequences of primers used in this study.**
(DOC)
