# Supplementary material for: Negative Regulation of Interferon-β Gene Expression during Acute and Persistent Virus Infections
Source: PLoS One. 2011 Jun 3;6(6):e20681. doi: 10.1371/journal.pone.0020681 (PMC3108996; doi:10.1371/journal.pone.0020681)
Supplement: Table S1 — Sequences of primers used in this study. (DOC) [file pone.0020681.s014.doc]

| **Table S1: Sequences of primers used in this study** | |
| --- | --- |
|  |  |
| **Gene name** | **Primer Sequences** |
| mouse IFNb | 5'-ccctatggagatgacggaga-3' and 5'-ctgtctgctggtggagttca-3' |
| mouse β-Actin | 5'-cctctatgccaacacagtgc-3' and 5'-acatctgctggaaggtggac-3' |
| SeV NP | 5'-gctcactcattagacacagataagcagcac-3' and 5'-gaaaagcggactcttgttgaccatagg-3' |
| mouse Stat1 | 5'-gaccacctctcttcctgtcg-3' and 5'-tgccaactcaacacctctga-3' |
| mouse IRF7 | 5'-tgcaaggtgtactgggaggt-3' and 5'-tcaccaggatcagggtcttc-3' |
| mouse Cxcl10 | 5'-tcatcctgctgggtctgagt-3' and 5'-ttttggctaaacgctttcatt-3' |
| human IFNb | 5'-gctgcagctgcttaatctcc-3' and 5'-tcctccaaattgctctcctg-3' |
| human GAPDH | 5'-ctgacttcaacagcgacacc-3' and 5'-ggtggtccaggggtcttact-3' |
